# Behavior Change Fast and Slow: Changing Multiple Key Behaviors a Long-Term Proposition?

**DOI:** 10.9745/GHSP-D-15-00331

**Published:** 2015-12-15

## Abstract

An intensive radio campaign in rural areas of Burkina Faso addressed multiple key behaviors to reduce child mortality, using a randomized cluster design. After 20 months, despite innovative approaches and high reported listenership, only modest reported change in behavior was found, mainly related to care seeking rather than habitual behavior such as hand washing. Various methodologic difficulties may have obscured a true greater impact. Analysis of the intervention after its full 35-month duration may reveal more impact, including on actual child mortality. Improving a number of key behaviors is essential to child survival efforts, and much of it may require strong and sustained efforts.

See related articles by Sarrassat and by Murray.

## SOME BEHAVIORS CHANGE QUICKLY

Human behavior is complex, often not completely rational, and profoundly influenced by social norms, structural constraints, opportunities, and habit. Yet we tend to approach behavior change interventions as discrete-in-time, “one-off” interventions. Of course some behaviors change remarkably readily. Think about the explosive adoption of cell phones globally. Or how use of plastic bags has plummeted in many jurisdictions in the United States (and in other countries), simply by adopting the “nudge” of levying a 5 cent charge on consumers.^1^

## BUT SOME BEHAVIORS CHANGE SLOWLY – THE CASE OF TOBACCO

Consider the reduction of smoking in the United States. It began with evidence emerging in the 1950s that led to a landmark US Surgeon General’s report in 1964.^2^ Efforts combatted tobacco industry assertions to try to deny and obscure the health effects and later were bolstered by recognition of tobacco as an addiction. Over the decades, evidence of the wide and varied harmful effects continued to mount. And when the harmful effects of secondhand smoke became recognized, it catalyzed a tipping point of strong social norms against smoking, since smoking could no longer be seen as harming only the smoker. All the while, public health initiatives ebbed and flowed. Rates of smoking declined steadily, but only gradually. A more recent breakthrough was targeting smoking prevention among adolescents. Thus, a package of interventions aimed at youth in New York City in the early 2000s combining higher cigarette taxes, prohibition of vending machines and tobacco sales near schools, strict enforcement of prohibition of sales to minors, and a very active mass media campaign produced a major drop in reported youth smoking.^3^ We need to recognize each behavior is different. Some such as those related to addiction and biologic drives can be particularly resistant to change.

## ADDRESSING MULTIPLE BEHAVIORS – THE INNOVATIVE *SATURATION+* INTERVENTION IN RURAL BURKINA FASO

Our global health community’s highly ambitious goals to end preventable child and maternal mortality^4^ mandate addressing a wide range of behaviors, both those that can be described as *habitual* (such as hand washing and proper nutrition) and *episodic* (for example, care seeking). Given the wide range of behaviors with major health consequences,^5^ it seems only practical to try to address them in some collective fashion and on a sustained basis, rather than as separate efforts.

Ending preventable child and maternal deaths mandates addressing a wide range of behaviors.

This issue of GHSP presents a paper on the implementation of such an ambitious approach using intensive mass media (radio) in Burkina Faso and its separate evaluation.^6^^,^^7^ The intervention was unusual in identifying rural communities served by virtually only one radio station, allowing the project to randomize 7 such areas to receive intensive mass media behavior change messaging and 7 not to receive it. The campaign messaging was directed to a range of behaviors modeled to have the most potential impact on child mortality based on extensive formative research. It was distinguished by innovative and practical implementation including free airtime from radio stations in return for training, equipment, and investment in solar power, as well as story-based messaging (using both short spots and longer dramas) based on local, rapidly developed content. Notably, it did not intervene to improve service delivery.

### Lukewarm Findings So Far

The separate midline evaluation (at 20 months) found a substantial proportion of the population reporting having heard the spots and dramas. And compared with the control areas, there were greater improvements in some key reported behaviors including saving money during pregnancy and care seeking/treatment for some conditions (diarrhea and possible pneumonia, two of the three leading causes of child death in Burkina Faso). But notably there were not significant comparative improvements in a variety of other behaviors including habitual behaviors such as hand washing or exclusive breastfeeding. Some support for impact came from a positive association between reported behavior change and exposure to “spot” messages though not to the dramas. And some corroboration of an effect on care seeking came from a marked relative increase in service utilization occurring in government primary care sites. The authors posit that care seeking may be easier to influence than habitual behaviors in this context, perhaps partly because onset of illness is more pressing and demanding of action. Conversely, habitual behaviors may be more entrenched and have cultural and structural limitations. It would be helpful to have some complementary qualitative evidence from the target population to see if their impressions were consistent with this hypothesis.

### Might the True Impact Actually Have Been Greater Than Measured?

Very possibly. The intervention area actually showed marked improvements in quite a number of behaviors, including *both* habitual and care seeking ones, but so too did the control areas (Table). So the evaluation properly compared the changes in both to see where improvements in the intervention area might be greater—a “difference-in-difference” analysis—which produced the more muted results. But actually the randomized allocation may not have ensured comparability of the intervention and control arms. There were only 7 clusters in each arm, and as it turned out there were important differences between the 2 arms. Intervention areas were poorer, had a higher proportion of Muslim population, and were farther from health facilities. While the evaluators adjusted for these measured factors in their analysis, such adjustment does not ensure that there is no remaining confounding, since unmeasured differences may remain. Moreover, it appears likely there could have been some “contamination” of messaging into the control areas; it was later learned that in one area there was overlap of radio coverage. Also, a fairly high proportion of those in the control areas reported they recognized spots when played to them (although it seems very possible this may reflect courtesy bias or confusion with other radio messages). Yet another source potentially attenuating a measured benefit were the numerous health-promoting activities carried out in both intervention and control areas by other health programs during this time, including a very successful malaria bed-net distribution program. Finally, the baseline and midline surveys were carried out in different seasons of the year. All of these factors contributed to “noise” that could possibly have washed out to some extent a true effect of the intervention.

**TABLE.** Attributes, Midline Findings, and Issues Associated With the Burkina Faso *Saturation+* Program

Attributes	Midline Findings	Issues
Randomized village clusters (7 intervention arms and 7 control arms)	Substantial listenership (as reported)	Substantial improvements in behavior seen in control areas
Areas believed to be isolated from other mass media messaging (outside the “electric grid”)	Improvement in some care seeking behaviors, but mostly no difference compared with control areas	Large differences between intervention and control areas at baseline: intervention areas poorer, more likely Muslim, and farther from health facilities
Mass media only (radio)	Some dose effect seen based on "spot" messages but not dramas	Seasonal difference between timing of baseline and midline surveys
Addressed multiple behaviors	Increase in service site utilization corroborates the reported increases in care seeking	Contamination of exposure to radio messages in one control area
Prioritized most important behaviors based on lives saved modeling		Various other health promoting activities occurred in both intervention and control areas
Extensive formative research		No qualitative data provided as yet to give further insights
Short spots and interactive dramas (story-based)		Mass media only
Aired multiple times per day (spots) or per week (dramas)		Many behaviors addressed; certain topics received more emphasis
Potential to reach others besides primary caregivers		No supply-side change limits service-related behavior
Local, rapidly developed, innovative content with quality control		More effect may take more time
Partnerships with local stations resulting in cost-efficiencies		
No efforts to increase or improve service delivery		

### What Else May Have Limited Impact?

First, this was a mass media-only intervention. Behavior change efforts through a combination of methods and channels have been found to be more effective in some settings. And those who are most disadvantaged and could benefit most from behavior change may have had the least exposure to the mass media. Second, addressing multiple behaviors may present some limitations. Clearly each behavior does not receive nearly as much emphasis as it would in an intensive single-behavior approach, and although the intervention changed the theme of the spots weekly, it’s possible multiple, different messages might be confusing to some listeners. Next, in order to have substantial increased utilization of primary health services, care seeking needs to be matched by availability and quality of those services. Although the local primary health system was able to respond to some extent to the increase in demand, its capacity to handle increased demand must have been constrained in this resource-poor environment. Lastly, it may simply be that in this context, some behaviors are inherently resistant to this kind of behavior change effort. Or more time might be needed. Fortunately, the intervention continued for another 15 months, ending in January 2015. The endline analysis should tell whether indeed further shift in behavior occurred, and to answer the ultimate question of whether there was an impact on actual mortality.

Behavior change efforts through a combination of methods and channels have been found to be more effective in some settings than mass media-only interventions.

Some behaviors may be inherently resistant to behavior change efforts or may require more time to change.

The good news though is that in both the intervention and the control areas, it appears that a number of key behaviors have been improving, perhaps in part because of the multiple other health intervention activities carried out in the overall area over time. It is useful to assess the impact of discrete behavior change interventions, but improving behavior over the long haul, probably resulting from multiple sources, seems the most reasonable approach for the most impact toward our global health goals. –*Global Health: Science and Practice*

Improving behavior over the long haul from multiple sources seems the most reasonable approach for the most impact.
